# Development of Digital Sampling for Spaceborne Fourier Transform Spectrometers Using Dual Reference Channel

**DOI:** 10.3390/s26072036

**Published:** 2026-03-25

**Authors:** Andrea Appiani, Diego Scaccabarozzi, Bortolino Saggin

**Affiliations:** 1 Department of Mechanical Engineering, Politecnico di Milano, 20156 Milan, Italy; andrea.appiani@polimi.it (A.A.); diego.scaccabarozzi@polimi.it (D.S.); 2 CISAS—Industrial Engineering Department, Università Degli Studi di Padova, 35131 Padua, Italy

**Keywords:** Fourier Transform Spectrometer, dual-reference processing, spaceborne instrumentation, mirror speed disturbance

## Abstract

This work presents an original implementation of the digital sampling pipeline for spaceborne Fourier Transform Spectrometers (FTSs). The implementation aims at improving the robustness of the spectrometer to harsh environmental conditions, including mechanical vibrations and a wide operational temperature range, avoiding the use of dedicated electronic hardware for the interferometer mirrors’ speed control and interferogram sampling. The FTS configuration is based on the constant time step sampling of the interferometer using a standard ADC (Analogue to Digital Converter), along with two metrology laser channels. The development tool is a MATLAB-based simulator developed to emulate the FTS and, in particular, the generation and acquisition of interferograms, incorporating harmonic vibrations and detector noise. The simulator was exploited to compare state-of-the-art techniques and newly implemented variants. An improvement of the arccosine method is first proposed, revising the normalisation process to exploit the full set of recorded data without discarding critical points. Subsequently, methods using two reference channels have been developed and evaluated. Two implementations are considered: two references at the same wavelength with an optimised phase shift (i.e., π/2) and two references at different wavelengths. Different data fusion strategies are compared in terms of spectral uncertainty, varying types of simulated disturbances and noise amplitudes. Results show that the optimal combination of two same-wavelength references consistently outperforms any other configuration, yielding lower average spectral errors and more stable performance over the frequency range and for a lower SNR of reference channels. Conversely, dual-wavelength strategies exhibit reduced accuracy, though they offer flexibility when fixed phase shifts cannot be maintained. The optimal combination of two same-wavelength reference channels, phase-shifted, is a promising configuration for spaceborne FTSs, so the development and test of an instrument breadboard is envisaged as the consequent development of this work.

## 1. Introduction

Fourier Transform Spectrometers (FTSs) are the instrument of choice for high-resolution spectral measurements in both ground-based and spaceborne applications, thanks to their multiplexing advantage and high throughput compared to dispersive spectrometers [1,2,3]. Recent advancements in frequency-comb-based FTS [4] and supercontinuum sources [5] have further demonstrated that software-only sampling pipelines can effectively mitigate high-frequency mechanical perturbations, eliminating the need for complex hardware stabilisation. In an FTS, the spectrum is retrieved from the interferogram, i.e., the intensity of the recombined beams as a function of the optical path difference (OPD). Under ideal conditions, a strictly constant mirror speed allows time-domain samples to be directly converted to OPD-domain samples without introducing artefacts [6,7].

In practice, however, and especially for spaceborne instruments, this assumption rarely holds. The most common sampling strategy, therefore, does not rely on constant time steps, but rather exploits a reference channel in the form of a monochromatic laser, injected into the interferometer alongside the incoming radiation and generating, through dedicated hardware, the sampling commands at the zero crossing (or multiple of), so ideally with equal OPD sampling [8,9]. Even the most recent FTS flown on space missions, such as the Infrared Atmospheric Sounding Interferometer (IASI) [10,11], the PFS onboard MarsExpress [12], the MER MiniTES [13] and the TIRVIM onboard ExoMARS [14], rely on this kind of sampling strategy.

Despite its advantages, the reference-channel approach is not immune to disturbances that can significantly degrade the interferometric data quality [15,16,17,18,19,20]. Mechanical perturbations are among the most critical, along with sampling step errors originating from various phenomena such as reference signal offsets, variations in mirror speed combined with acquisition channels’ different phase delays, and cyclic misalignments of optical components [21,22]. These effects are particularly relevant in spaceborne instruments, where vibration insulation systems, commonly used in ground-based configurations, are often not feasible because of mass and volume limitations. A variety of vibration and shock sources may be present onboard spacecraft and rovers: moving mechanisms, thermal fluctuations, electromagnetic forces, and even fluidic effects [23,24,25]. Their combined action has been shown to produce significant spurious signatures in interferometric measurements [26]. It must be noticed that even among space instruments, there is a significant difference between Earth Observation (EO) instruments, like IASI and CrIS, where the mass budget allows for interface vibration insulation systems, and those devoted to planetary exploration. In the field of planetary observation, the mass limitation is so stringent that interface dampers, if present, are mostly devoted to limiting the stresses on the optical components during launch phases while, during operation, the instruments must deal with the micro vibration environment. This explains why EO FTS rarely report mechanical vibration disturbance; conversely, for instance, the Planetary Fourier Spectrometer (PFS) onboard Mars Express [13] highlighted how mechanical micro-vibrations can induce sampling jitters that degrade spectral quality even with a disturbance amplitude below 1% of the nominal OPD speed [15].

The resulting disturbances can lead to a condition in which, even with a reference channel, the interferogram is not sampled at constant OPD steps. Moreover, cyclic misalignments may induce amplitude modulations in the interferogram, altering the instrument’s modulation function and compromising spectral accuracy and resolution if not properly corrected in post-processing.

Two main avenues can address these challenges: (i) improving the opto-mechanical design to mitigate vibrations and misalignments and (ii) developing data-processing techniques to achieve constant OPD sampling intervals. As anticipated, since spaceborne applications mostly prevent the implementation of vibration insulation systems or additional hardware, enhanced data-processing algorithms represent a particularly attractive route to improve performance without penalising the instrument’s resources.

This work focuses on advancing data processing for spaceborne FTS and presents novel developments and extensions of previously developed methods. The challenge is to develop the equal time sampling technique to the point of making the FTS tolerant to vibration disturbances up to levels in the range of 50% of the nominal OPD speed and reliable enough to replace the state-of-the-art sampling system for space instruments based on dedicated hardware. Specifically, this includes a revised normalisation scheme for the arccosine method to exploit the full data record and the exploitation of two reference channels rather than one. The choice of this dual-reference geometry is further supported by recent studies on path-dependent vibration sensitivity [27], which suggest that multi-channel metrology provides superior common-mode rejection of mechanical jitter compared to single-channel systems. These approaches are validated through a dedicated MATLAB-based simulator emulating spaceborne conditions, including speed disturbances and electrical noise.

The paper is organised as follows. Section 2 reviews the state of the art in OPD correction and highlights the main limitations. Section 3 introduces the proposed processing methods, including the modified arccosine approach and the dual-reference strategies. Section 4 presents the numerical results under various disturbance and noise scenarios. Finally, Section 5 discusses the findings and outlines recommendations for future spaceborne FTS implementations.

## 2. OPD Methods Reconstruction and Velocity Correction

The digital sampling strategies mentioned in Section 1 can be broadly grouped into three families, each with specific strengths and limitations regarding their applicability to spaceborne instruments.

### 2.1. Zero Crossings Methods: Brault’s Algorithm

Brault’s method, developed in the mid-1990s, allows OPD reconstruction without closed-loop control of the moving mirrors. It relies on the timing of specific features in a monochromatic reference laser interferogram, such as zero-crossings, to resample the main interferogram from the time domain to the OPD domain [28,29,30]. Interpolation between fringe events allows the retrieval of the OPD at evenly spaced points.

While conceptually simple, this method has limitations in spaceborne applications. This digital resampling approach mirrors techniques currently used in Optical Frequency Domain Reflectometry (OFDR), where auxiliary interferometers calibrate nonlinear frequency sweeps through digital phase reconstruction [31]. Furthermore, modern implementations have refined this for fibre-fed systems to specifically correct scan-induced jitter [32]. It requires additional hardware to detect and store zero-crossings, which increases system mass. Interpolation errors can arise when fringe events occur unevenly, especially under high-frequency mechanical disturbances. Furthermore, significant oversampling of the reference signal is necessary to achieve acceptable accuracy [33,34].

### 2.2. Phase Demodulation Methods: Campbell and Hilbert Transform Approaches

Phase demodulation methods avoid the need for event timers by extracting the OPD directly from the reference interferogram. Campbell’s method uses quadrature demodulation to reconstruct the instantaneous phase [35,36], reducing hardware complexity and avoiding interpolation errors. The Hilbert transform approach reconstructs the analytic signal of the interferogram and derives the phase without requiring prior knowledge of the reference frequency [37,38].

However, both approaches have intrinsic limitations. The maximum correctable disturbance frequency is constrained by the reference channel, and high-frequency components beyond this limit cannot be corrected. Physically, these limitations lead to a quantifiable degradation of spectral resolution and SNR under velocity jitter [32]. Campbell’s method is sensitive to disturbances near the reference frequency due to the low-pass filtering, while the Hilbert transform approach, although less sensitive to such filtering effects, is more prone to noise amplification under high-frequency jitter. In both cases, correction errors increase with high levels of mirror speed disturbances, resulting in artefacts such as ‘integration ghosts’ [39] and spectral distortions resulting from sampling irregularities [40].

### 2.3. Direct Phase Computation Methods

The arccosine method represents a direct phase computation approach that overcomes some limitations of demodulation-based techniques. Unlike Campbell’s or Hilbert transform methods, whose correction bandwidth is limited by the reference-channel frequency, the arccosine method computes the phase directly from the normalised interferogram, allowing correction of higher-frequency mirror speed disturbances [41,42,43].

For a monochromatic source of wavelength λ, the reference interferogram S(t) produced by a Michelson interferometer is a pure cosine function of the OPD (or time) [4,5]:

(1) S(t)=Acos(4πσvt)

where σ is the wavenumber (i.e., 1/λ), v is the mirror velocity, and A is a constant proportional to the optical power of the monochromatic source and to the interferometer throughput. Since the arccosine function is defined over [−1, 1], the interferogram must be normalised prior to phase extraction. To this end, the analytic signal associated with the reference interferogram S(t) is computed as:

(2) $$S_{H}\left(t\right)=S\left(t\right)+iH\{S\left(t\right)\}$$

where H{·} denotes the Hilbert transform. The amplitude envelope is then obtained as the modulus of the analytic signal:

(3) $$A\left(t\right)=\left|S_{H}\left(t\right)\right|$$

which is subsequently low-pass filtered (e.g., using a Butterworth filter, 4th order, cutoff frequency 10 Hz) in order to remove residual high-frequency components and isolate low amplitude variations due to laser power fluctuations or finite bandwidth effects. The normalised signal $I_{n}\left(t\right)$ is finally defined as:

(4) $$I_{n}\left(t\right)=S\left(t\right)/A\left(t\right)$$

and used for direct phase extraction through the arccosine operation.

Even after normalisation, some points may exceed the [−1, 1] interval; these must be discarded to avoid singularities in the arccosine inversion, which also helps reduce noise amplification near the extrema of the interferogram. The wrapped phase is then computed as:

(5) $$\phi_{w}\left(t\right)=\arccos\left(I_{n}\left(t\right)\right),\;\phi_{w}\in \left[0,\;\pi \right]$$

to resolve the inherent ambiguity in the sign of the phase, a quadrature sign (QS) is computed from the Hilbert transform of the normalised interferogram:

(6) $$\mathrm{QS}\left(t\right)=\mathrm{sign}\left(H\left[I_{n}\left(t\right)\right]\right)$$

**Figure 1:**
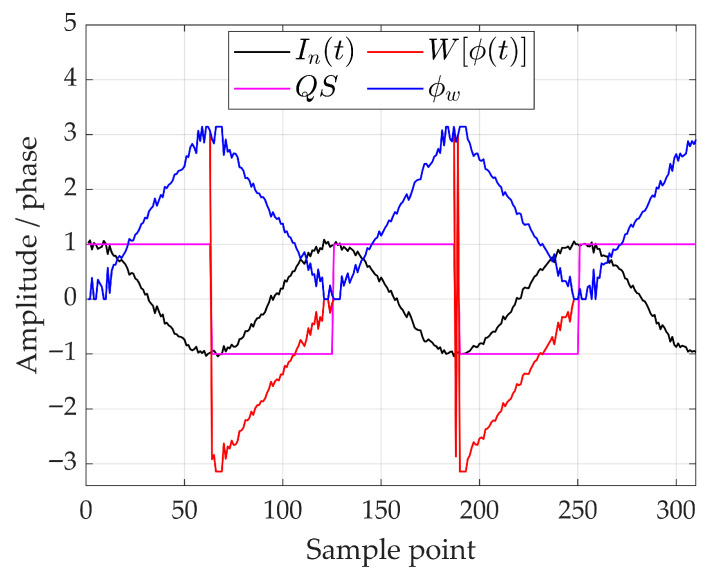
Factors involved in OPD calculation with the arccosine method.

Multiplying $\phi_{w}$ by QS(t) produces the signed wrapped phase W[ϕ(t)], which can then be unwrapped to obtain the continuous phase ϕ(t). A representation of all the relevant quantities involved in the computation of the OPD by the arccosine method is shown in Figure 1, displaying: the normalised interferogram $I_{n}\left(t\right)$, the phase $\phi_{w}$, the signum QS and the wrapped phase W[ϕ(t)]. Among the listed correction methods, the arccosine one has proved to be the best performing for a wide range of both amplitude of the acting mirror speed disturbance as well as noise level present on the reference channel [44].

Figure 1 highlights the physical necessity of the quadrature sign QS(t) to distinguish mirror velocity reversals during vibrations. Without this directional information, velocity fluctuations would lead to fringe-counting errors, causing a total loss of spectral coherence.

The sensitivity of the arccosine method to noise is governed by its derivative:

(7) $$\frac{d}{dI_{n}}\arccos\left(I_{n}\right)=-\frac{1}{\sqrt{1-I_{n}^{2}}}$$

This shows that near the extrema of the interferogram ($\left|I_{n}\right|\to 1$), the derivative becomes very large, amplifying even small noise contributions, which makes phase estimation highly sensitive. Conversely, near the zero crossings ($I_{n}\approx 0$), the derivative is minimal, and the method is robust against noise. The quadrature sign QS provides additional guidance, indicating that the phase is most reliable where QS≈1 (zero crossings) and least reliable where QS→0 (extrema of the interferogram).

**Figure 2:**
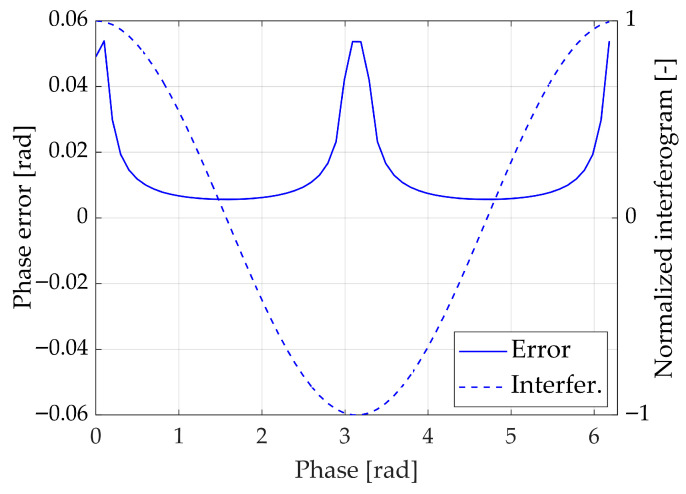
Error over a period of monochromatic interferogram with a 40 dB SNR.

To quantitatively verify these theoretical considerations, Monte Carlo simulations (e.g., 10^4^ iterations) were performed. In these simulations, synthetic interferograms were generated with additive Gaussian noise at controlled signal-to-noise ratios (ranging from 40 dB to 20 dB), and the phase was extracted using the arccosine method. The resulting phase errors were collected as a function of the normalised interferogram values. The simulations confirmed that errors are concentrated near the extrema, whereas regions around zero crossings remain highly stable; this concept can be visualised in Figure 2, where a 40 dB SNR noise is considered.

These results provide a direct validation of both the derivative-based sensitivity analysis and the role of the quadrature sign in guiding robust phase extraction.

Building on this, the observed noise sensitivity motivates the introduction of multi-channel extensions, in which a second reference interferogram is used to provide complementary phase information. By combining information from two interferograms that exhibit different sensitivity profiles along the signal, regions affected by noise in one channel can be compensated by more stable regions in the other, leading to a smoother and more robust phase reconstruction.

## 3. Methods and Tools

A numerical simulator of an FTS was developed in MATLAB (R2022b) to assess the performance of state-of-the-art and novel OPD reconstruction methods, comparing the capability of correcting velocity errors. The tool enables the generation of synthetic interferograms while introducing both deterministic velocity disturbances and additive noise, thus providing a controlled environment to test the robustness of different processing approaches.

The simulator allows the user to define all relevant parameters of the acquisition process, such as the mean velocity of the moving mirror, the frequency and amplitude of a superimposed monoharmonic disturbance, the acquisition frequency, the signal-to-noise ratio, and the wavelengths of the reference channels. The OPD is computed by integrating the prescribed velocity profile, and the corresponding interferograms are generated for both the reference channel and the scientific inputs. White Gaussian noise can be added to reproduce electrical disturbances, with the signal-to-noise ratio adjusted to the desired level.

**Figure 3:**
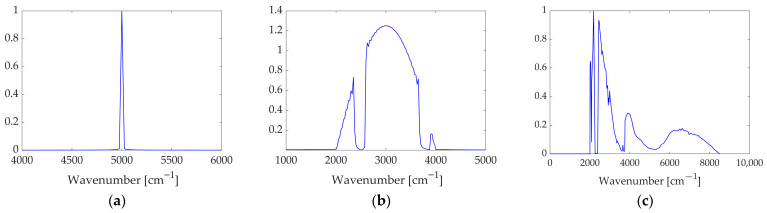
Generated main input radiation: (**a**) monochromatic, (**b**) broadband with simulated absorption bands and (**c**) Mars-representative spectra.

In addition to the reference channel, the simulator generates interferograms of the main input radiation. Three types of spectral inputs are considered: a monochromatic source, a synthetic broadband source with predefined absorption features, and a Mars-representative spectrum [13]. This setup enables a comprehensive assessment of the correction algorithms across diverse and progressively more realistic scenarios. All three spectra are represented in Figure 3. Unless otherwise specified, the numerical results presented in the following Sections refer to the Mars-representative spectrum, which provides the most realistic test case in terms of spectral complexity, while the remaining results (associated with the monochromatic and broadband spectrum) are reported in Appendix A and Appendix B.

Two established velocity correction techniques were implemented as benchmarks, i.e., the Hilbert transform method and the standard arccosine method. The new methods were then introduced and compared against these baselines. Once the OPD is retrieved, interferograms are resampled over an evenly spaced OPD grid and transformed into spectra via Fast Fourier Transform (FFT), using cubic interpolation for resampling.

To quantify the performance of the different approaches, a normalised mean root square error (NMRSE) was defined, comparing the retrieved spectra with the corresponding ideal ones generated internally by the simulator. Lower NMRSE values indicate better reconstruction accuracy. The index is calculated as follows:

(8) $$\mathrm{NMRSE}=\frac{100\sqrt{\sum_{i=1}^{n}{\left(A_{\mathrm{calc},\;i}-A_{\mathrm{id},\;i}\right)}^{2}/n}}{A_{\mathrm{id},\;\max}}$$

$A_{\mathrm{calc},\;i}$ and $A_{\mathrm{id},\;i}$ represent the amplitude of the i-th spectral line of the corrected and ideal spectrum, respectively; n is the number of spectral lines, which depends on the number of elements of the regular OPD vector over which the interferograms are resampled, while $A_{\mathrm{id},\;\max}$ is the maximum amplitude in the ideal spectrum and is used to normalise the error.

Concerning the parameters used for the simulations, these have been set to replicate the acquisition by means of a miniaturised spaceborne spectrometer [45,46,47] and are reported in Table 1, including the mirror average speed v_0_, sampling frequency f_s_, duration of the simulation t and wavelength of the reference channel λ_ref_.

A parametric study was carried out by introducing a single-frequency (monoharmonic) velocity disturbance in the simulated mirror motion. The disturbance frequency was varied across independent simulation runs, spanning the range from 10 Hz to 1 kHz in steps of 10 Hz, while keeping the disturbance harmonic in each individual case. The selection of this frequency range was motivated by the need to encompass the main mechanical disturbance sources typically encountered in spaceborne platforms. In particular, disturbances associated with reaction wheel assemblies are commonly reported in the range of approximately 50–350 Hz, while higher-frequency components related to Inertial Measurement Units (IMU) and similar subsystems may extend up to about 500–650 Hz [48]. Extending the analysis up to 1 kHz ensures that the full bandwidth of relevant platform-induced disturbances is covered, while also allowing the investigation of potential higher-frequency effects on the interferometric signal.

Two disturbance amplitudes, corresponding to 20% and 60% of the mean mirror velocity, were considered. In addition, white Gaussian noise was added at two levels (SNR = 40 dB and SNR = 20 dB) to emulate electrical noise in the measurement chain. This framework enables a systematic assessment of the robustness and limitations of both established and newly proposed correction methods under controlled disturbance conditions.

### 3.1. Modified Normalisation in the Arccosine Method

The standard arccosine method relies on a normalisation step in which the reference interferogram is divided by its analytic signal, which is low-pass filtered with a Butterworth filter. A known drawback is that, after normalisation, some samples can still fall outside the [−1, 1] interval, preventing the inversion through the arccosine function. The conventional approach discards those points, resulting in a loss of information and possible artefacts during the interpolation of the interferogram on a regular OPD grid.

To overcome this issue, a modified normalisation procedure was implemented. Instead of discarding points exceeding the cosine domain, their phase was slightly perturbed by adding a small random component, ensuring that the interferogram could still be unwrapped and resampled without inconsistencies. The perturbation was dimensioned to be one order of magnitude smaller than the errors already introduced by electrical noise, thus negligible in terms of overall accuracy.

The new procedure was tested against the standard method under four representative combinations of speed disturbance amplitude and noise level. The average NMRSE obtained throughout the considered bandwidth with the modified normalisation was consistently lower, as reported in Table 2.

Although the numerical improvement in the error metric is relatively modest, it should be noted that the error value, as introduced earlier in Equation (8), depends on the number of spectral lines over which it is evaluated. In this context, the observed reduction remains meaningful. Moreover, the modified normalisation exhibited a more stable behaviour and prevented the loss of samples. For these reasons, it was adopted as the baseline arccosine implementation in the subsequent analyses.

### 3.2. Single-Wavelength Methods

As discussed in Section 2, one of the main weaknesses of the standard arccosine method lies in the inversion of the cosine function near the extrema of the interferogram, where the derivative of the arccosine diverges, and noise amplification becomes severe. Figure 2 shows that phase errors are strongly concentrated in these regions, whereas the areas around zero crossings remain considerably more robust.

To mitigate this issue, the use of a second reference channel was investigated. Assuming the adoption of an additional interferogram generated at the same wavelength, the relative phase shift remains constant along the entire signal, making the identification of an optimal shift crucial. This was addressed by analysing the phase error distributions obtained numerically as introduced earlier: by taking two of these error functions, shifting one with respect to the other, and calculating the root mean square of their point-by-point sum, the optimal configuration can be determined. This analysis revealed that a phase shift of π/2 minimises the combined error. In practice, this means that the maxima and minima of one interferogram coincide with the zero crossings of the other, so that when one signal operates in its most noise-sensitive region, the other remains in a more stable regime.

Three different data fusion strategies were then explored to exploit the complementary information provided by the two interferograms.

#### 3.2.1. Substitution Strategy

The first approach, referred to as substitution, consists of building a single combined phase by alternating portions of the two unwrapped phases. The basic principle is to exploit the fact that, for two interferograms generated at the same wavelength and shifted by π/2, the extrema of one correspond to the zero crossings of the other, as reported in Table 3.

This approach ensures that, when one channel contains a noisy region, the other provides a phase segment less sensitive to noise. To implement it, the first step is to identify the characteristic points of each interferogram: maxima, minima, and zero crossings. For this purpose, a moving average filter is applied to the signum QS, the phase $\phi_{w}$, and the normalised interferogram $I_{n}$. This produces the corresponding averaged quantities, $\bar{\mathrm{QS}}$, $\bar{\phi }$, and $\bar{I_{n}}$. The filter uses a window of 10 points, roughly 1/6 of the interferogram period under the nominal simulator parameters (see Table 1). Table 4 lists the criteria applied to these averaged quantities to define maxima, minima, and zero crossings.

**Figure 4:**
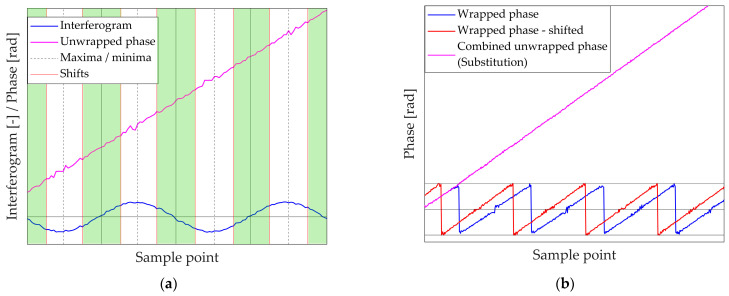
(**a**) Shift points on a single reference interferogram; (**b**) combination by substitution resulting phase.

Once the characteristic points are identified, their mutual correspondence is established by averaging their positions across the two interferograms, exploiting the π/2 shift. This strategy leverages the spatial complementarity of the signals, ensuring the sampling clock is always derived from high-slope regions. The resulting set of averaged positions is then exploited to derive transition points, defined halfway between consecutive extrema and zero crossings (vertical red lines in Figure 4a), so that the combined phase includes only the low-scattering portions of each channel (green highlighted regions in Figure 4a). This effectively eliminates the metrology ‘blind spots’ (extrema) where the SNR is lowest.

This procedure yields a combined phase that is continuous and free from the irregular scattering typically observed near interferogram stationary points. As shown in Figure 4b, the resulting unwrapped phase (magenta) alternates contributions from the two channels while avoiding the irregular portions clearly visible in the individual phases (blue and red). In this way, interpolation on the OPD grid and subsequent spectral reconstruction can be carried out without artefacts, ensuring improved robustness of the processing chain.

#### 3.2.2. Linear Weight Strategy

A second data fusion strategy for combining the information from the two reference channels is based on a linear combination of the two unwrapped phases. In this approach, the contribution of each interferogram varies gradually according to a weight function, rather than switching sharply as in the substitution method. The combined phase $\phi_{\mathrm{lin}}$ is computed as:

(9) $$\phi_{\mathrm{lin}}=w\phi_{1}+\left(1-w\right)\phi_{2}$$

where w is a linear varying weight, ranging from 0 to 1. The definition of the weight function relies again on the identification of characteristic points of each interferogram: maxima, minima, and zero crossings (both rising and falling). Regions near zero crossings are assigned higher weights because they are less sensitive to noise, while regions close to stationary points receive lower weights. This ensures that each interferogram contributes more strongly to the segments where its phase estimate is most reliable.

The first step of the procedure is therefore analogous to the substitution strategy: maxima, minima, and zero crossings are detected using the criteria outlined in Table 4, and their correspondences across the two channels are determined as in Table 3. Next, the positions at which the weight function w assumes the boundary values 0 and 1 are set according to Table 5.

Intermediate weight values are then obtained by linear interpolation between these points, producing a smooth transition across the interferogram.

**Figure 5:**
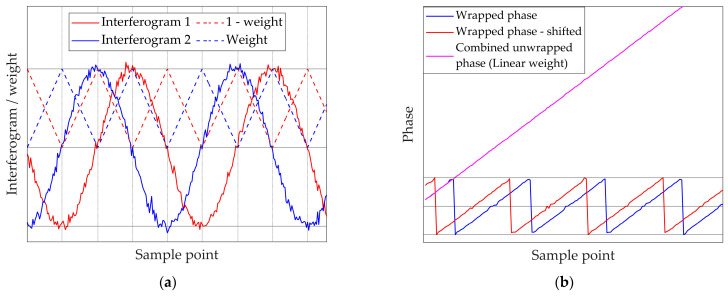
(**a**) Phase-shifted interferograms and associated weight functions; (**b**) combination by linear weighting resulting phase.

A visualisation of this procedure is reported in Figure 5a. For the first interferogram (blue), vertical lines indicate its maxima, minima, and zero crossings. The corresponding linear weight w is shown as a dashed blue line. For completeness, a phase-shifted second interferogram is included, along with its complementary weight 1−w (continuous and dashed red lines, respectively). Figure 5 illustrates the transition from a discrete ‘hard-switching’ logic to a continuous signal fusion; physically, the overlapping weights represent a weighted trust model between the two sensors.

The resulting linear combination, represented in Figure 5b, yields a smoothly blended phase that preserves robustness to noise, reduces discontinuities, and avoids abrupt transitions. This is significant because, in a real spaceborne FTS, any sharp numerical discontinuity in the reconstructed OPD would be mathematically indistinguishable from a real mechanical shock, thus generating ‘ghost’ spectral artefacts.

This linear weighting approach provides a flexible alternative to the substitution strategy. It can accommodate variations in the signal-to-noise ratio between the two channels and allows gradual blending of contributions, rather than hard selection of stable segments. The smooth transition shown here ensures that the spectral baseline remains clean, preserving the instrument’s ability to detect weak absorption lines and improving the overall stability of the reconstructed phase while maintaining robustness in noise-sensitive regions.

#### 3.2.3. Variance Minimisation

A third approach for combining the phases from two reference channels is based on variance minimisation. The goal is to construct a linear combination of the two interferometric phases that minimises the expected variance of the resulting phase, producing the most reliable estimate in the presence of noise. Assume two monochromatic interferometric channels affected by independent noise:

(10) $$S_{1}=\cos\left(\theta \right)+n_{1}$$

(11) $$S_{2}=\cos\left(\theta +\pi /2\right)+n_{2}$$

where $n_{1}$ and $n_{2}$ are independent, zero-mean Gaussian noise terms with variances $\sigma_{1}^{2}$ and $\sigma_{2}^{2}$, respectively. The corresponding phases are extracted using the arccosine method:

(12) $$\phi_{1}=\arccos\left(S_{1}\right)$$

(13) $$\phi_{2}=\arccos\left(S_{2}\right)$$

The combined phase is defined as a linear combination:

(14) $$\phi_{\mathrm{opt}}=a\phi_{1}+b\phi_{2}$$

with weights a and b chosen to minimise the variance of ϕ_opt_. For independent variables, the variance of a linear combination is:

(15) $$\mathrm{Var}\left(\phi_{\mathrm{opt}}\right)=a^{2}\mathrm{Var}\left(\phi_{1}\right)+b^{2}\mathrm{Var}\left(\phi_{2}\right)$$

To account for the nonlinear transformation introduced by the arccosine function, a first-order Taylor expansion is applied:

(16) $$\mathrm{Var}\left(\arccos\left(S\right)\right)\approx {\left(\frac{\mathrm{darccos}}{\mathrm{dS}}\right)}^{2}\;\mathrm{Var}\left(S\right)$$

and using:

(17) $$\frac{d}{\mathrm{dS}}\arccos\left(S\right)=-\frac{1}{\sqrt{1-S^{2}}}$$

the variances of the individual phases become:

(18) $$\mathrm{Var}\left(\phi_{1}\right)\approx \frac{\sigma_{1}^{2}}{\left(1-S_{1}^{2}\right)}$$

(19) $$\mathrm{Var}\left(\phi_{2}\right)\approx \frac{\sigma_{2}^{2}}{\left(1-S_{2}^{2}\right)}$$

Substituting the expression for Var($\phi_{\mathrm{opt}}$):

(20) $$\mathrm{Var}\left(\phi_{\mathrm{opt}}\right)=a^{2}\frac{\sigma_{1}^{2}}{\left(1-S_{1}^{2}\right)}+b^{2}\frac{\sigma_{2}^{2}}{\left(1-S_{2}^{2}\right)}$$

Minimising this variance with respect to a and b under the constraint a+b=1 yields the optimal weights:

(21) $$a=\frac{1-S_{1}^{2}}{2-S_{1}^{2}-S_{2}^{2}}$$

(22) $$b=\frac{1-S_{2}^{2}}{2-S_{1}^{2}-S_{2}^{2}}$$

This procedure can be applied directly to the interferogram samples themselves, without requiring a fixed phase shift between the channels. The resulting combined phase automatically downweighs the noisy extrema (where |S|→1) and emphasises regions where each channel is more robust, i.e., near zero crossings. Compared to substitution or linear weight strategies, this approach is fully adaptive and minimises the expected phase error for every OPD sample, providing a statistically optimal combination. The first-order Taylor approximation is adopted to ensure computational efficiency and to enable the derivation of a closed-form analytical solution for the optimal weights. This approach is particularly suited for real-time implementations where mathematical tractability is essential. Given the high sampling-to-signal frequency ratio, the truncation error is expected to be dominated by the noise floor. This choice is validated by the numerical results discussed in Section 4: even under high noise levels and large phase deviations induced by velocity disturbances, the method remains robust and continues to outperform alternative strategies, confirming that the approximation error does not significantly impact the total error budget.

**Figure 6:**
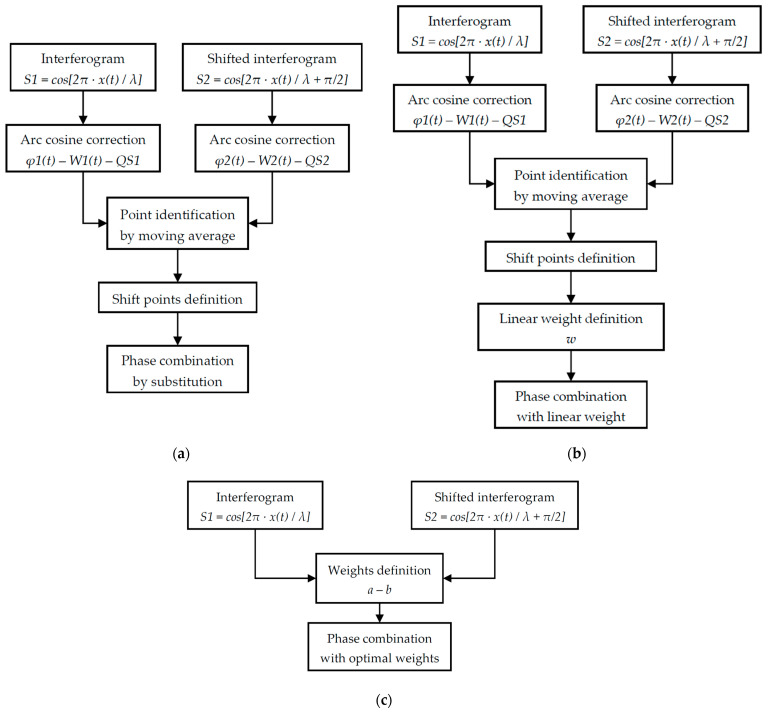
Flowcharts for the three different envisioned single-wavelength data fusion strategies: combination by (**a**) substitution, (**b**) linear weighting and (**c**) variance minimisation.

A schematic summary of the three data fusion strategy procedures is given in Figure 6a, b and c for the substitution, linear weight and variance minimisation techniques, respectively.

### 3.3. Dual Wavelength Method

After the investigation of the strategies based on a single reference wavelength, the case of combining interferograms obtained at different wavelengths has been considered. In this situation, a constant phase shift between the two channels cannot be defined across the entire acquisition, since the relative phase difference between signals of distinct optical frequencies varies continuously with the optical path difference.

**Figure 7:**
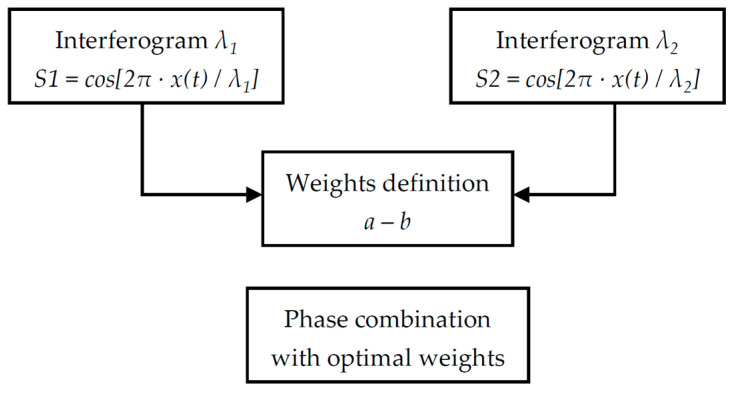
Flowchart for phase combination exploiting reference channels with two different wavelengths.

Among the three methods previously described, the only one that can be extended without critical limitations to the dual-wavelength case is the minimum variance phase combination strategy, based on variance minimisation. In fact, this approach relies on the point-by-point computation of the optimal weights (Equations (21) and (22)), which are defined locally on the interferograms and therefore do not require a fixed phase relationship between the reference channels. This property makes the method intrinsically suitable for the fusion of signals at different wavelengths. A summary of the dual wavelength method is reported in the flowchart in Figure 7.

A set of five wavelengths in the visible and near-infrared domain was selected for the analysis, corresponding to values commonly available in commercial laser sources (1064, 670, 635, 532 and 405 nm). From these, ten possible dual-wavelength pairings can be constructed (Table 6). For each case, the minimum variance phase has been calculated using the previously introduced formulation.

The same simulation framework employed for the single-wavelength analysis has been adopted, with consistent values of velocity disturbance, signal amplitude, and additive noise. The dual-wavelength variance minimisation strategy has then been compared to the three reference single-wavelength strategies (substitution, linear weighting, and variance minimisation). For consistency, a wavelength of 635 nm has been chosen for the single-wavelength cases.

## 4. Numerical Results

In this Section, the performance of the proposed data fusion strategies is assessed and compared with reference methods from the state of the art. The evaluation is carried out by computing the NMRSE on reconstructed spectra, considering different disturbance conditions. As previously mentioned, two amplitudes of speed fluctuation (20% and 60% of v_0_) and two noise levels (SNR = 20 dB and SNR = 40 dB) were analysed, leading to four test cases.

While the absolute NMRSE values depend on the specific input spectrum, the relative performance ranking among the considered methods was observed to remain unchanged for identical disturbance and noise conditions. On this basis, the performance plots reported in the following are limited to the Martian representative case, while the remaining results (for monochromatic and broadband spectra cases) are provided in Appendix A and Appendix B for completeness.

The horizontal axis of the performance plots reports the frequency of the speed disturbance in Hz, while the vertical axis shows the NMRSE as defined in Equation (8). For clarity, the results are also summarised in the following tables, which report the mean NMRSE across all disturbance frequencies for each test case and for each considered spectrum.

### 4.1. Methods Performance Assessment

**Figure 8:**
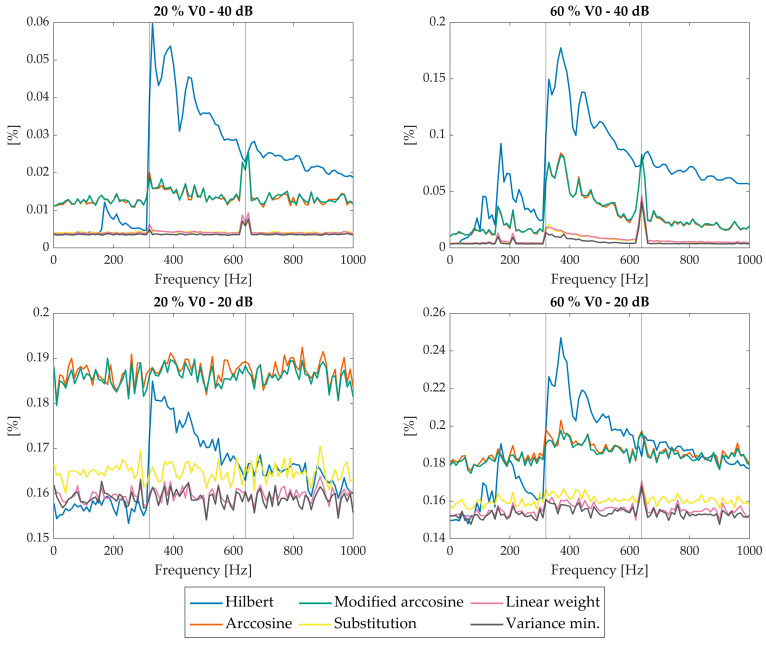
NMRSE trends for different correction strategies (single-wavelength methods–635 nm) at different noise and disturbance amplitude levels, with respect to the Martian spectrum. X-axis reports disturbance frequency.

Computed NMRSEs for the benchmark and new OPD reconstruction methods, considering the Martian spectrum, are reported in Figure 8 and summarised in Table 7. Summaries of the results for the monochromatic and broadband spectra are listed in Table 8 and Table 9, while the associated NMRSE trends are reported in Appendix A.

**Figure 9:**
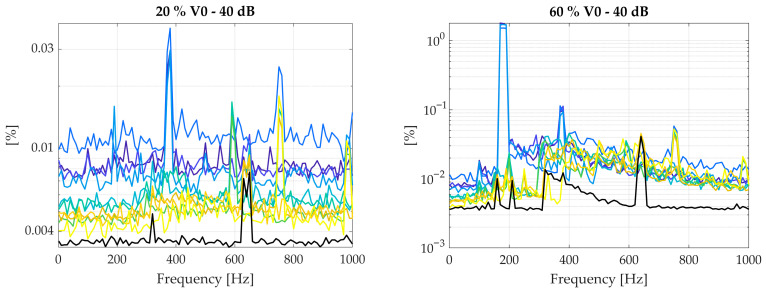

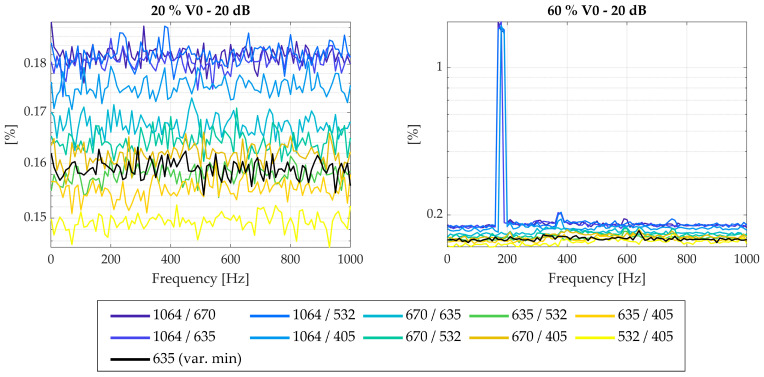
NMRSE for dual-wavelength—variance minimisation combination correction strategy for different wavelength combinations. X-axis reports disturbance frequency.

Figure 9 shows trends of the NMRSE for the ten dual-wavelength combinations; the average NMRESs of the entire frequency range are summarised in Table 10. For a clearer quantitative comparison, Table 10 also includes the single-wavelength reference case at 635 nm. The results for the monochromatic and broadband spectra are reported in Table 11 and Table 12, and their associated NMRSE diagrams can be found in Appendix B.

The single-wavelength method was also tested at 405 nm, i.e., the shortest available reference. The performance metrics comparing the additional case with the double-wavelength best combination are reported in Table 13, Table 14 and Table 15 for the Martian, monochromatic and broadband spectrums, respectively. For all tables, the worst and best achieved NMRSE values are highlighted in red and green, respectively.

**Figure 10:**
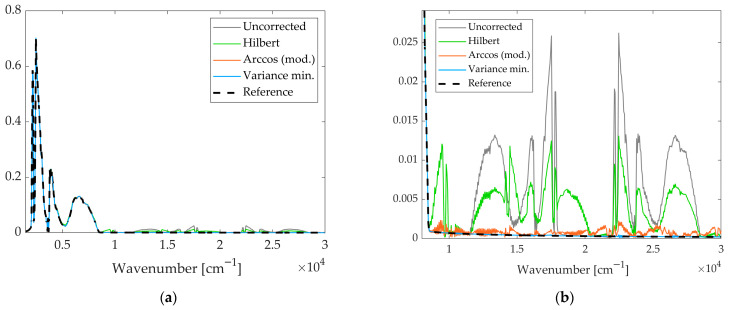
Comparison of Mars-representative spectra obtained from simulations with a 60% v0 amplitude speed disturbance at 400 Hz, processed using different correction strategies together with the uncorrected and ideal spectra. (**a**) Full spectral range. (**b**) Zoomed view of the frequency region affected by ghost spectral components.

Mars-representative spectra obtained from simulations considering a 60% v0 amplitude speed disturbance at 400 Hz, processed using the reference and new methods are shown in Figure 10.

### 4.2. Discussion

Results in Figure 8 show that, on average, the modified arccosine method outperforms the Hilbert transform, which also exhibits irregular trends and sharp jumps at the reference frequency $f_{\mathrm{ref}}=v_{0}/\lambda$ and its harmonics. These instabilities are not desirable and confirm the limitations of Hilbert-based phase methods.

The proposed fusion strategies, namely substitution, linear weighting, and variance minimisation combination, based on delayed single wavelength, were evaluated using two reference interferograms at 635 nm shifted by π/2. The optimal combination based on the variance minimisation method consistently achieves the lowest NMRSE across all scenarios, as shown in Figure 8 and Table 7, Table 8 and Table 9. Substitution and linear weighting methods also yield improvements over the benchmarks, with linear weighting generally performing slightly better than substitution, although in some configurations the opposite occurs. The hierarchy among single-wavelength methods is preserved in every case (monochromatic, broadband and Martian representative spectra), with the variance minimisation approach representing the most robust and accurate solution.

Besides the NMRSE results, the effectiveness of the correction methods can be further assessed through a visual inspection of Figure 10a,b. These figures compare Mars-representative spectra obtained from simulations considering a 60% v_0_ amplitude speed disturbance at 400 Hz, processed using different strategies, namely the Hilbert method, the modified arccosine approach, and their combination through variance minimisation, together with the unprocessed case. The effect of the 400 Hz perturbation is clearly visible in the uncorrected spectrum as pronounced ghost spectral components. Physically, high-frequency velocity jitter modulates the interferogram, shifting energy into frequency regions where the ideal Mars-representative signal (dashed line) is null. As can be observed, the uncorrected spectrum exhibits pronounced ghost spectral components in frequency regions where the ideal spectrum is expected to be flat and equal to zero. The application of correction methods progressively mitigates these artefacts, in agreement with the trends highlighted by the NMRSE values. In particular, the spectra processed via variance minimisation show a clear reduction in the amplitude of the spurious components when compared to the other correction strategies.

The analysis was then extended to the case where reference interferograms at two different wavelengths are combined. Five wavelengths were considered (1064, 670, 635, 532, and 405 nm), leading to ten possible dual-wavelength combinations. As previously noted, only the variance minimisation strategy was applied. As shown by the obtained results, the performance of the dual-wavelength variance minimisation strategy depends strongly on the selected pair. Indeed, a clear trend emerges: combinations involving shorter wavelengths systematically achieve lower NMRSE values, while those involving longer wavelengths are characterised by larger errors and more irregular behaviours, with abrupt jumps near the reference frequencies. The best-performing pair was found to be consistently the 532 nm/405 nm combination, which, in some cases (notably at 20 dB SNR), even outperforms the single-wavelength strategy at 635 nm, as shown in Table 10, Table 11 and Table 12 for the couple 532–405 nm.

Eventually, a performance comparison between the best dual-wavelength combination and the single-wavelength methods at 635 nm and 405 nm, summarised in Table 13, Table 14 and Table 15, confirmed that decreasing the wavelength improves the robustness of the spectral reconstruction. Most importantly, the single-wavelength optimal combination with 405 nm clearly outperforms all dual-wavelength combinations, including the best-performing 532 nm/405 nm case. This behaviour is consistent across all disturbance, noise and considered spectra conditions, demonstrating that exploiting the shortest available wavelength in a single-wavelength optimal strategy remains the most effective solution.

The selection of the reference wavelengths (1064, 670, 635, 532, and 405 nm) was driven by the availability of high-stability commercial sources. Notably, the algorithm proved robust even for the 1064–532 nm pair, which presents an integer 2:1 ratio. While such a ratio could theoretically lead to simultaneous stationary points if the signals are perfectly in phase, the inherent presence of additive noise and the discrete nature of the sampling prevent persistent numerical singularities. Furthermore, the 532–405 nm pair achieved the best performance by combining a non-integer ratio (~1.31), which introduces a beneficial phase sliding effect, with the higher metrological resolution inherent to shorter wavelengths.

## 5. Conclusions

This work presented novel data processing methodologies for spaceborne Fourier Transform Spectrometers (FTS) aimed at improving robustness and accuracy under realistic operating conditions. A dedicated numerical simulator was developed to emulate the acquisition of interferograms, including both deterministic speed disturbances (vibration disturbance on the FTS) and additional noise, simulating measurement chain disturbances. Through this framework, several correction methods were systematically evaluated on monochromatic, broadband and Mars-like inputs.

The analysis first demonstrated that a revised normalisation of the classical arccosine method enables the full exploitation of the acquired data, eliminating the need to discard critical regions and improving overall spectral accuracy. Building on this baseline, new dual-reference approaches were introduced. The quantitative assessment confirmed that the proposed variance minimisation strategy, using two same-wavelength references (635 nm) shifted by π/2, consistently achieves the highest accuracy. For a Mars-representative spectrum under 40 dB SNR and 20% speed disturbance, this approach yielded an average NMRSE of 0.0037, marking an 83% error reduction compared to the Hilbert transform benchmark (NMRSE = 0.0221). Even in the most severe tested conditions (20 dB SNR and 60% velocity disturbance), the variance minimisation method maintained superior robustness with an NMRSE of 0.1538.

Dual-wavelength combinations were also examined, showing that shorter reference wavelengths yield improved accuracy due to a reduced phase error sensitivity. Specifically, moving from a 635 nm to a 405 nm reference laser further reduced the average NMRSE from 0.0037 to 0.0025 in moderate conditions, and from 0.1538 to 0.1281 in the harshest scenario. However, the single-wavelength optimal combination applied to the shortest available wavelength outperformed all the others, demonstrating that exploiting the most stable and least noise-sensitive phase information remains the most effective approach across all disturbance levels and spectral contents.

The numerical validation was extended to a Mars-representative input spectrum, selected as a realistic and challenging case for assessing the robustness of the proposed methods under conditions representative of planetary observation scenarios. Results confirmed the same hierarchy observed for monochromatic and broadband spectra, indicating that the relative performance of the different processing strategies does not depend on the spectral content of the input signal.

Overall, the results indicate that the optimal combination of two same-wavelength references, shifted by the appropriate phase value, represents a reliable baseline for future spaceborne FTS implementations.

While the current results establish a solid theoretical and algorithmic foundation, future work will focus on the experimental validation of the proposed algorithms using laboratory interferometers under controlled perturbations. This upcoming campaign aims at bridging numerical and experimental domains, assessing the end-to-end impact on reconstructed spectra and verifying the performance of the dual-reference architecture in a physical test-bed environment.

## Figures and Tables

**Table 1:** FTS simulator main parameters.

Mirror Avg. Speed	Sampling Freq.	Sim. Duration	Ref. Wavelength
$v_{0}$	$f_{s}$	t	$\lambda_{\mathrm{ref}}$
0.2 mm/s	20 kHz	10 s	635 nm

**Table 2:** NMRSE comparison between the standard and modified arccosine methods.

	20% $v_{0}$ 40 dB	60% $v_{0}$ 40 dB	20% $v_{0}$ 20 dB	60% $v_{0}$ 20 dB
Standard	0.0068	0.0173	0.0612	0.0647
Modified	0.0067	0.0151	0.0588	0.0611

For the selected case: Best NMRSE–Worst NMRSE.

**Table 3:** Correspondence among interferograms’ points.

Interferogram	Shifted Interferogram
Maximum	Falling zero crossing
Minimum	Rising zero crossing
Rising zero crossing	Maximum
Falling zero crossing	Minimum

**Table 4:** Criteria for points identification, based on the relevant parameters of the moving average of relevant parameters.

Type of Point	Criteria
Maximum	$\{\begin{array}{c} \bar{\mathrm{QS}}=0\\ \bar{\phi }<1.2\;rad \end{array}$
Minimum	$\{\begin{array}{c} \bar{\mathrm{QS}}=0\\ \bar{\phi }>\pi -1.2\;rad \end{array}$
Zero crossings	$-0.1<\bar{I_{n}}<0.1$

**Table 5:** Interferogram-weight points correspondence.

Interferogram	Weight
Minimum	0
Rising zero crossing	1
Maximum	0
Falling zero crossing	1

**Table 6:** Adopted wavelength combinations for the dual wavelength method.

		Longest [nm]			
		1064	670	635	532
Shortest [nm]	670	X			
	635	X	X		
	532	X	X	X	
	405	X	X	X	X

**Table 7:** NMRSE average value over the entire frequency range for the different correction strategies (single-wavelength methods–635 nm) with respect to the case studies presented in Figure 8.

Method	20% $v_{0}$ 40 dB	60% $v_{0}$ 40 dB	20% $v_{0}$ 20 dB	60% $v_{0}$ 20 dB
Hilbert	0.0221	0.0707	0.1648	0.1860
Arccosine	0.0135	0.0289	0.1871	0.1860
Modified arccosine	0.0136	0.0293	0.1860	0.1849
Substitution	0.0042	0.0074	0.1648	0.1606
Linear weight	0.0042	0.0078	0.1596	0.1555
Variance minimisation	0.0037	0.0056	0.1590	0.1538

For the selected case: Best NMRSE–Worst NMRSE.

**Table 8:** NMRSE average value over the entire frequency range for the different correction strategies (single-wavelength methods–635 nm) with respect to the monochromatic spectrum.

Method	20% $v_{0}$ 40 dB	60% $v_{0}$ 40 dB	20% $v_{0}$ 20 dB	60% $v_{0}$ 20 dB
Hilbert	0.0122	0.0386	0.0522	0.0666
Arccosine	0.0061	0.0149	0.0580	0.0603
Modified arccosine	0.0061	0.0149	0.0575	0.0596
Substitution	0.0018	0.0034	0.0507	0.0500
Linear weight	0.0018	0.0037	0.0491	0.0484
Variance minimisation	0.0016	0.0025	0.0490	0.0480

For the selected case: Best NMRSE–Worst NMRSE.

**Table 9:** NMRSE average value over the entire frequency range for the different correction strategies (single-wavelength methods–635 nm) with respect to the broadband spectrum.

Method	20% $v_{0}$ 40 dB	60% $v_{0}$ 40 dB	20% $v_{0}$ 20 dB	60% $v_{0}$ 20 dB
Hilbert	0.0407	0.1313	0.2648	0.3005
Arccosine	0.0229	0.0502	0.2903	0.2921
Modified arccosine	0.0230	0.0506	0.2894	0.2911
Substitution	0.0070	0.0123	0.2624	0.2572
Linear weight	0.0069	0.0132	0.2544	0.2499
Variance minimisation	0.0061	0.0095	0.2537	0.2475

For the selected case: Best NMRSE–Worst NMRSE.

**Table 10:** NMRSE average value calculated over the entire frequency range for all of the possible reference wavelength combinations presented in Figure 9.

Wavelengths [nm]	20% $v_{0}$ 40 dB	60% $v_{0}$ 40 dB	20% $v_{0}$ 20 dB	60% $v_{0}$ 20 dB
1064–670	0.0088	0.0692	0.1818	0.2205
1064–625	0.0085	0.0677	0.1806	0.2075
1064–532	0.0120	0.0665	0.1820	0.2207
1064–405	0.0077	0.0665	0.1753	0.1997
670–625	0.0061	0.0139	0.1675	0.1651
670–532	0.0061	0.0143	0.1642	0.1613
670–405	0.0051	0.0124	0.1580	0.1555
625–532	0.0053	0.0127	0.1617	0.1586
625–405	0.0053	0.0133	0.1552	0.1530
532–405	0.0050	0.0136	0.1494	0.1478
Single (635)	0.0037	0.0056	0.1587	0.1545

For the selected case: Best NMRSE–Worst NMRSE.

**Table 11:** NMRSE average value over the entire frequency range for all of the possible reference wavelengths with respect to the monochromatic spectrum.

Wavelengths [nm]	20% $v_{0}$ 40 dB	60% $v_{0}$ 40 dB	20% $v_{0}$ 20 dB	60% $v_{0}$ 20 dB
1064–670	0.0038	0.0331	0.0567	0.0799
1064–625	0.0036	0.0330	0.0563	0.0720
1064–532	0.0053	0.0323	0.0575	0.0795
1064–405	0.0033	0.0323	0.0544	0.0700
670–625	0.0028	0.0077	0.0517	0.0521
670–532	0.0026	0.0073	0.0504	0.0507
670–405	0.0024	0.0066	0.0484	0.0486
625–532	0.0025	0.0070	0.0497	0.0499
625–405	0.0022	0.0064	0.0476	0.0477
532–405	0.0021	0.0061	0.0455	0.0458
Single (635)	0.0016	0.0025	0.0490	0.0483

For the selected case: Best NMRSE–Worst NMRSE.

**Table 12:** NMRSE average value over the entire frequency range for all of the possible reference wavelengths with respect to the broadband spectrum.

Wavelengths [nm]	20% $v_{0}$ 40 dB	60% $v_{0}$ 40 dB	20% $v_{0}$ 20 dB	60% $v_{0}$ 20 dB
1064–670	0.0136	0.1177	0.2854	0.3615
1064–625	0.0139	0.1205	0.2838	0.3352
1064–532	0.0190	0.1189	0.2842	0.3580
1064–405	0.0129	0.1209	0.2762	0.3287
670–625	0.0109	0.0267	0.2654	0.2626
670–532	0.0095	0.0249	0.2589	0.2568
670–405	0.0091	0.0224	0.2493	0.2474
625–532	0.0098	0.0248	0.2556	0.2534
625–405	0.0088	0.0232	0.2442	0.2430
532–405	0.0082	0.0219	0.2348	0.2343
Single (635)	0.0061	0.0094	0.2536	0.2485

For the selected case: Best NMRSE–Worst NMRSE.

**Table 13:** Average NMRSE comparing variance minimisation strategy for optimally shifted single-wavelength cases (635 nm and 405 nm) and 532–405 nm dual-wavelength combination, processing Martian spectrum (Figure 3c).

Wavelength(s) [nm]	20% $v_{0}$ 40 dB	60% $v_{0}$ 40 dB	20% $v_{0}$ 20 dB	60% $v_{0}$ 20 dB
635	0.0037	0.0056	0.1590	0.1538
532–405	0.0050	0.0136	0.1494	0.1478
405	0.0025	0.0040	0.1281	0.1259
Best	405 nm	405 nm	405 nm	405 nm

For the selected case: Best NMRSE–Worst NMRSE.

**Table 14:** Average NMRSE comparing variance minimisation strategy for optimally shifted single-wavelength cases (635 nm and 405 nm) and 532–405 nm dual-wavelength combination, processing monochromatic spectrum (Figure 3a).

Wavelength(s) [nm]	20% $v_{0}$ 40 dB	60% $v_{0}$ 40 dB	20% $v_{0}$ 20 dB	60% $v_{0}$ 20 dB
635	0.0016	0.0025	0.0490	0.0480
532–405	0.0021	0.0061	0.0455	0.0458
405	0.0011	0.0018	0.0388	0.0387
Best	405 nm	405 nm	405 nm	405 nm

For the selected case: Best NMRSE–Worst NMRSE.

**Table 15:** Average NMRSE comparing variance minimisation strategy for optimally shifted single-wavelength cases (635 nm and 405 nm) and 532–405 nm dual-wavelength combination, processing broadband spectrum (Figure 3b).

Wavelength(s) [nm]	20% $v_{0}$ 40 dB	60% $v_{0}$ 40 dB	20% $v_{0}$ 20 dB	60% $v_{0}$ 20 dB
635	0.0061	0.0095	0.2537	0.2475
532–405	0.0082	0.0219	0.2348	0.2343
405	0.0041	0.0073	0.2020	0.2007
Best	405 nm	405 nm	405 nm	405 nm

For the selected case: Best NMRSE–Worst NMRSE.

**Figure A1:**
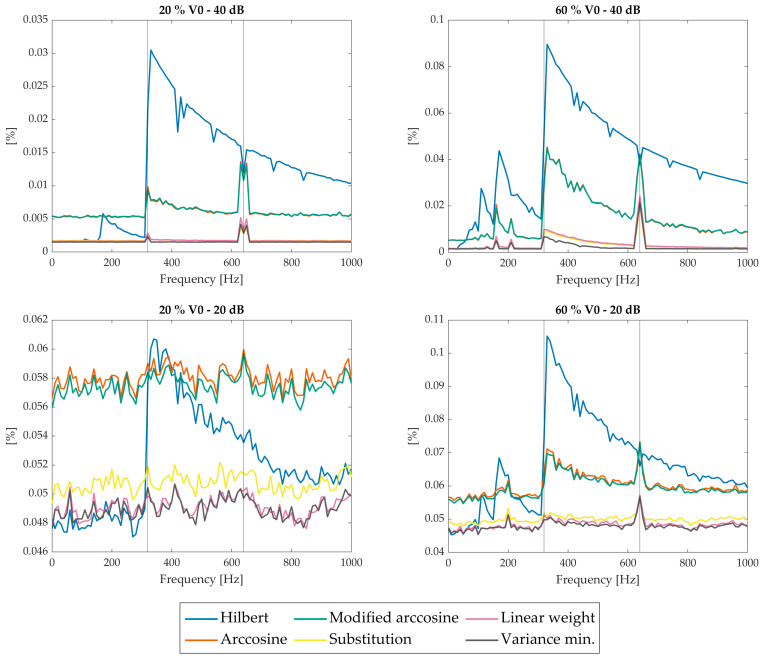
NMRSE trends for different correction strategies (single-wavelength methods–635 nm) at different noise and disturbance amplitude levels, referred to the monochromatic spectrum. X-axis reports disturbance frequency.

**Figure A2:**
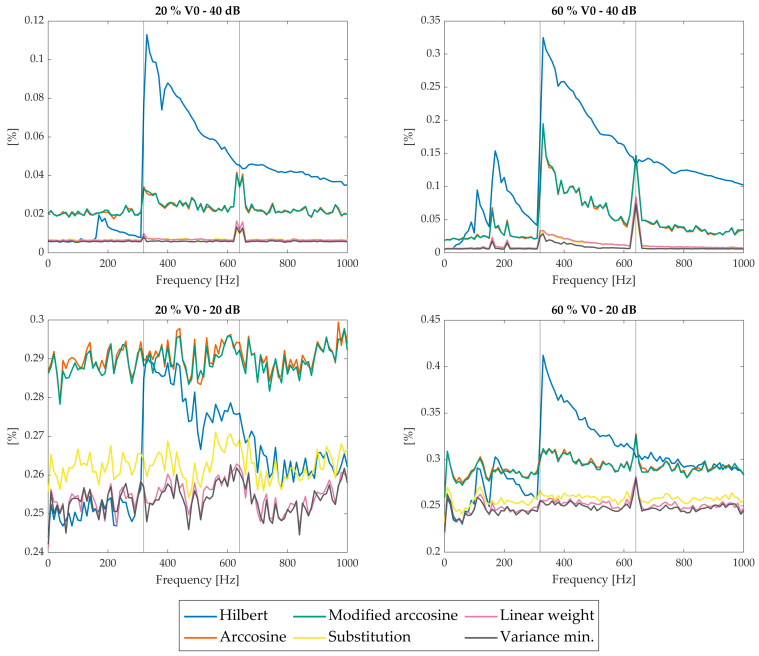
NMRSE trends for different correction strategies (single-wavelength methods–635 nm) at different noise and disturbance amplitude levels with respect to the broadband spectrum. X-axis reports disturbance frequency.

**Figure A3:**
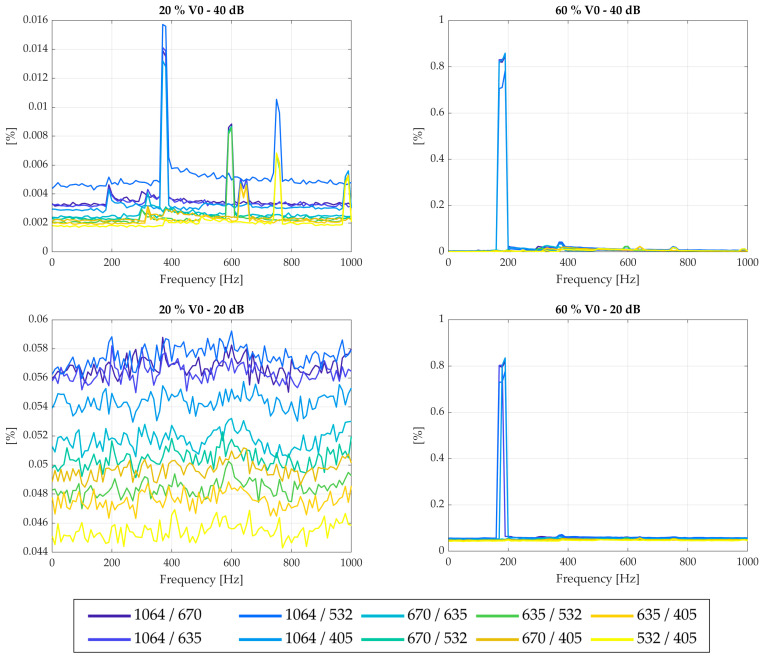
NMRSE for dual-wavelength—optimal combination correction strategy for different wavelength combinations, with respect to the monochromatic spectrum. X-axis reports disturbance frequency.

**Figure A4:**
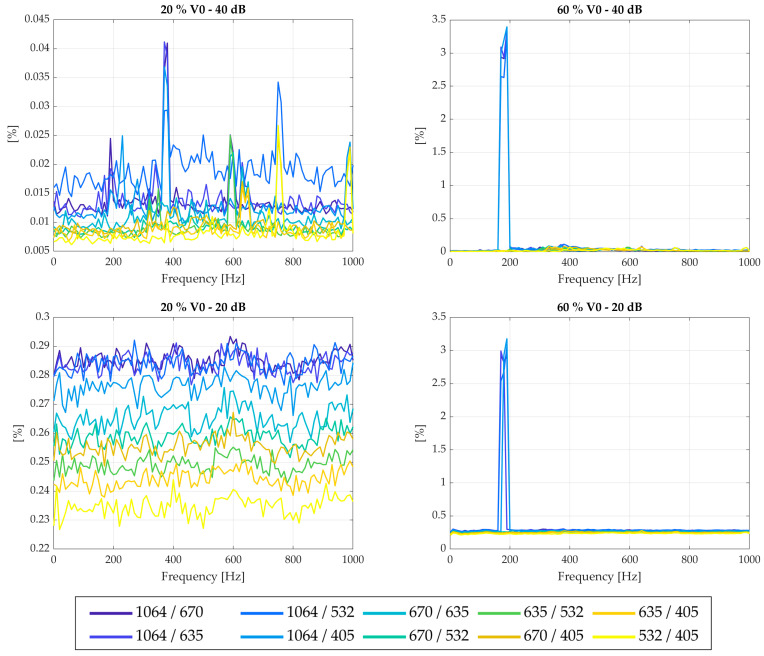
NMRSE for dual-wavelength—optimal combination correction strategy for different wavelength combinations, with respect to the broadband spectrum. X-axis reports disturbance frequency.

## Data Availability

Dataset available on request.

## Abbreviations

The following abbreviations are used in this manuscript:

FTS	Fourier Transform Spectrometer
OPD	Optical path difference
FFT	Fast Fourier Transform
NMRSE	Normalised mean root square error
SNR	Signal-to-noise ratio
